# Using overbooking to manage no-shows in an Italian healthcare center

**DOI:** 10.1186/s12913-018-2979-z

**Published:** 2018-03-15

**Authors:** Chiara Anna Parente, Domenico Salvatore, Giampiero Maria Gallo, Fabrizio Cipollini

**Affiliations:** 1 IRCCS SDN, Napoli, Via E. Gianturco 113, 80143 Naples, Italy; 2 Department of Accounting, Management and Economics, University of Naples Parthenope, Via Generale Parisi, 13, 80132 Naples, Italy; 3 Corte dei conti, Sezione regionale di controllo per la Lombardia, via Marina 5, 20121 Milan, Italy; 4 Department of Statistics, Informatics and Applications (DiSIA) G. Parenti, University of Florence, Viale Giovanni Battista Morgagni, 59, 50134 Florence, Italy

**Keywords:** No-show, Overbooking, Healthcare, Logistic regression, Simulation, Scheduling

## Abstract

**Background:**

In almost all healthcare systems, no-shows (scheduled appointments missed without any notice from patients) have a negative impact on waiting lists, costs and resource utilization, impairing the quality and quantity of cares that could be provided, as well as the revenues from the corresponding activity. Overbooking is a tool healthcare providers can resort to reduce the impact of no-shows.

**Methods:**

We develop an overbooking algorithm, and we assess its effectiveness using two methods: an analysis of the data coming from a practical implementation in an healthcare center; a simulation experiment to check the robustness and the potential of the strategy under different conditions. The data of the study, which includes personal and administrative information of patients, together with their scheduled and attended examinations, was taken from the electronic database of a big outpatient center. The attention was focused on the Magnetic Resonance (MR) ward because it uses expensive equipment, its services need long execution times, and the center has actually used it to implement an overbooking strategy aimed at reducing the impact of no-shows. We propose a statistical model for the patient’s show/no-show behavior and we evaluate the ensuing overbooking procedure implemented in the MR ward. Finally, a simulation study investigates the effects of the overbooking strategy under different scenarios.

**Results:**

The first contribution is a list of variables to identify the factors performing the best to predict no-shows. We classified the variables in three groups: “Patient’s intrinsic factors”, “Exogenous factors” and “Factors associated with the examination”. The second contribution is a predictive model of no-shows, which is estimated on context-specific data using the variables just discussed. Such a model represents a fundamental ingredient of the overbooking strategy we propose to reduce the negative effects of no-shows. The third contribution is the assessment of that strategy by means of a simulation study under different scenarios in terms of number of resources and no-show rates. The same overbooking strategy was also implemented in practice (giving the opportunity to consider it as a quasi-experiment) to reduce the negative impact caused by non attendance in the MR ward. Both the quasi-experiment and the simulation study demonstrated that the strategy improved the center’s productivity and reduced idle time of resources, although it increased slightly the patient’s waiting time and the staff’s overtime. This represents an evidence that overbooking can be suitable to improve the management of healthcare centers without adversely affecting their costs and the quality of cares offered.

**Conclusions:**

We shown that a well designed overbooking procedure can improve the management of medical centers, in terms of a significant increase of revenue, while keeping patient’s waiting time and overtime under control. This was demonstrated by the results of a quasi-experiment (practical implementation of the strategy in the MR ward) and a simulation study (under different scenarios). Such positive results took advantage from a predictive model of no-show carefully designed around the medical center data.

**Electronic supplementary material:**

The online version of this article (10.1186/s12913-018-2979-z) contains supplementary material, which is available to authorized users.

## Background

Patient’s non–attendance, defined as “missing a scheduled appointment without canceling it”, is an important issue for the management of healthcare centers. Although reported no-show rates vary widely, from a minimum of 3% to a maximum of 80% [1], depending on the type of facility and practice [2, 3], a patient’s non–attendance to scheduled appointments may affect productivity, consume resources, prolong the waiting time for an examination and reduce customer satisfaction. These economic effects have direct clinical counterparts, because poorly managed no-shows interrupt continuity and quality of cares [4], delay diagnoses and treatments of other patients and compromise the intention of health companies to invest in new technological and human resources.

A stream of research investigated the factors explaining the propensity of patients to no-show. The list of the possible explanatory variables include:

1. factors related to individuals, such as demographic characteristics, ethnicity, type of medical insurance, socioeconomic conditions, disease-related factors (i.e. acute vs. chronic disease), the past history in terms of previous reservations, no-shows and cancellations [2];
2. environmental conditions, such as office accessibility, difficulties in reaching the healthcare center, lack of transportation, lack of child care, lead time of examination, examination type and service quality [2, 5–7];
3. practices designed to increase patient’s attendance rates, as education, penalties and reminders by phone or email [5, 8–11].

Early works on patient’s non–attendance assumed homogeneous patients, i.e. sharing the same no-show probability [12]; more recent contributions allowed such a probability to be different across patients, so as to estimate it based on their specific characteristics and historical data [13–15]. Some appointment scheduling techniques discussed in existing literature assumed a deterministic and fixed service time [4], while others considered it as random, for example exponentially [12, 14, 15] or gamma [13] distributed. These approaches aimed at identifying which time of the day an examination should be booked, restricting the freedom of choice of the patients [14, 16]. Such scheduling procedures were frequently associated with overbooking (defined as multiple-booking of patients in a common time slot) aimed at increasing the number of patients the healthcare center could receive. As reported in [4], overbooking practices were more convenient when the no-show rate was high, the healthcare center served a larger number of patients and the service time variability was low, with the aim of finding an optimal compromise between expected additional patients and the costs of patients waiting time and staff overtime [4, 14–16].

This aim of this paper is to develop a context-specific overbooking algorithm and to assess its effectiveness using two methods: 1) the analysis of real data coming from the implementation of the algorithm in an healthcare center and 2) a simulation experiment to examine the robustness and potential of the strategy under different conditions.

At the best of our knowledge, our study is one of the first proposing an algorithm for overbooking in healthcare centers, also implemented in practice. The only overbooking algorithm we found was proposed by [4].

Our algorithm is novel because:

1. It considers different levels of no-show and a different number of available MR scanners;
2. It simulates the variables show/no-show, actual duration and patient being early/late;
3. It is actually implemented in the MR ward of the healthcare center.

The healthcare center, located in Southern Italy is a leading provider of medical imaging and laboratory diagnostic services. It operates within the Italian National Health Service (NHS) and with health insurance companies, offering many diagnostic procedures structured in different wards.

Italy’s healthcare system provides universal coverage largely free of charge at the point of delivery. Public funding is collected at the national level through general taxation and then distributed to 21 regions, which are in charge of making most administrative decisions on the organization of healthcare. In turn, regional administrations deliver services within their area through health districts, which may be in charge of some public health decisions and collaborate with local municipalities on health and public assistance matters.

Care is provided by public, not for profit and private providers (such as the healthcare center in this study) accredited by the public health service which funded 78.2% of overall health-care spending in 2012 [17]. Public providers may be independent public hospitals or organizational units of health districts.

The catchment area identifies the healthcare center as important in the region, as it includes patients from mostly the province of location, but also a few from the neighboring provinces and even from the neighboring regions. The healthcare center only provides ambulatory medical care diagnostic procedures and it does not provide hospital (inpatient) care nor primary care services. Patients have access to the procedures in the healthcare center only if referred by a primary care doctor or a by a specialist doctor; each referral should be accompanied by the medical indication for the diagnostic procedure ordered. Some of the diagnostic procedures available for outpatients in this specific healthcare center may be complex and not available even in big secondary care hospitals.

## Methods

### Data

All the data in this study were taken from the electronic database of this big outpatient healthcare center specialized in diagnostic procedures. The database includes personal and administrative information of the patients together with all their scheduled and attended examinations.

The healthcare center accepts reservations through its call center or its website and charges the costs of the procedures to the patient only after the execution of the examination and only in case there is any amount not covered by the NHS or by patient’s insurance. Therefore, there is no direct financial loss for a non-attending patient. As noted, because of the regional regulation, the healthcare center cannot perform the examinations considered in this study unless an external specialist medical doctor has prescribed it, in order to guarantee the appropriateness of the procedure.

The reference population of this study included all patients booking an examination from January 1, 2012 to December 31, 2014 for a total of 104,188 patients and 152,547 examinations. During this interval, the average rate of no-show was 14.6%; 73.8% of patients attended their booked examinations, 16.2% were no-show only once and the remaining 10% had more than one no-show. The study included 45.8% males and 54.2% females with an average age, respectively, of 52 and 53.

### Analysis

The main purpose of this work is to develop and estimate a model to predict whether a booked examination may result in a no-show. This tool helps the center in its overbooking strategy to reduce the fall in revenue caused by non–attending patients, while not increasing the patient’s waiting time and the overtime of medical staff. The potential explanatory variables (defined in Table 1 and homogeneously classified in Table 2) represent characteristics of the individuals (for example, their age or a previous no-show), factors associated with the examination (for example if it involved the use of a contrast agent or its price) and exogenous factors (for example date and time of the appointment or the weather forecast at that day). The base levels for the categorical variables are reported in Table 3. We performed the analysis separately for each department of the healthcare center (Echography, Echo Doppler, Mammography, Orthopantomography, Radiography, Computer-Assisted Tomography – CT, and Magnetic Resonance - MR); in what follows, we focused on the latter, because it has long execution time and needs expensive equipment; moreover, since the healthcare center has later implemented an overbooking strategy, this gave us the opportunity to evaluate the effectiveness of such a strategy in areal setting. Models estimated by ward differ just by the explanatory variables, with MR and CT sharing the same list, while in the other wards the Contrast Agent is excluded, as not needed. Moreover, Mammography did not include Gender and excluded the youngest 0–18 age group (one patient, excluded in agreement with the recommendations that the execution of that examination “not before age 25” [18]). Orthopantomography did not include the Price of the Examination because there was a fixed price for all patients.

**Table 1:** Definition of the variables included in the analyses

Variable	Description
No-show	Missing a scheduled appointment without canceling it. When a patient cancelled his reservation, in fact, this was removed just from the dependent variable because a cancelled appointment could not be classified as a no-show because the slot was made available for another patient.
Insurance status	In Italy, the majority of the examinations is covered by the NHS, but there is a portion that is privately paid by patients • NHS: Examination covered by the NHS • Private: Examination paid directly by the patient or by a private insurance
Rate of previous cancellations	Number of cancellations divided by number of reservations in previous examinations of the booked patient since 2012 in any ward
Rate of previous no-shows	Number of no-show divided by number of reservations in previous examinations of the booked patient since 2012 in any ward
Booking confirmation	An automated phone service calls patients aged over 65 2 days before the appointment to get a confirmation • Not Confirmed: if the patient did not confirm the appointment • Confirmed: if the patient confirmed the appointment
Type of booking	Method of reservation used by patients • Phone: reservation by phone with the assistance of an operator • Web: reservation through the institutional website (active since April 2014)
Time of the day	MR and CT wards had an opening time ranging from 6 AM to 2 AM (+1d); all remaining wards had opening time 8 AM - 8 PM. Accordingly, we considered the following time of the day: • 6 AM–8 AM: for MR and CT wards only • 8 AM-1 PM: for all wards • 1 PM–8 PM: for all wards • 8 PM-2 AM (+1d): for MR and CT wards only
Long weekend	It indicated whether the day of the appointment fell in a week with a public holiday • Yes: week with a public holiday • No: week without a public holiday
Weather forecast	An automated system downloads every day from the web (http://api.wunderground.com) the weather forecasts of the same and of the following 3 days • Clear: day without rain or storm • Rain: day with rain • Storm: day with a storm
Text message reminder service	Two days before the examination, an automated service sends a text message to all patients aged between 18 and 65 (active since April 2014) • Not yet activated: examinations booked before April 2014 • Activated but not sent: examinations booked since April 2014 without sending a text message reminder because the system did not know the patient mobile phone number or because he/she was over 65 • Sent: examination booked after April 2014 with text message reminder sent
No NHS coverage period	In Italy, the NHS covers most of the examinations until a cap on the NHS budget was reached, causing a stop in the coverage. This happened, usually, in the last weeks of the year • No: examination booked in the coverage period • Yes: examination booked when there was no NHS insurance because the budget was spent in full
Price of the examination	Price paid by the patient. It was set to zero for the examinations covered by NHS and to the whole price otherwise
Waiting list	Number of days between the booking and the examination
Time allowed	Expected duration of the examination
Hourly revenue	The revenues of all examinations divided by the product between the number of working hours and the number of active MR scanners
Waiting time	Difference between the time when the patient starts his/her examination and the latest between the scheduled time of the appointment and the arrival time at the healthcare center
Idle time	Idle time of the scanners across the appointments
Overtime	Staff overtime at the end of the day

**Table 2:** Descriptive statistics

Variable	Total	No-Show	Show	Test statistic
*Patient’s intrinsic factors*				
Gender				20.56 ^***^
Male	45.78%(35,152)	45.78 (35152)	46.11%(30,374)	
Female	54.22%(41,629)	56.23%(6137)	53.89%(35,492)	
Age group				224.26 ^***^
0–18	4.26%(3268)	4.56%(498)	4.21%(2770)	
19–45	35.2%(27,030)	40.35%(4404)	34.35%(22,626)	
46–64	37.64%(28,901)	36.78%(4014)	37.78%(24,887)	
65–79	20.31%(15,597)	15.9%(1736)	21.04%(13,861)	
80+	2.59%(1985)	2.41%(263)	2.61%(1722)	
Insurance status				10.68 ^**^
NHS	86.31%(66,270)	87.31%(9530)	86.14%(56,740)	
Private	13.69%(10,511)	12.69%(1385)	13.86%(9126)	
Rate of previous cancellations	0.21 ± 0.26	0.19 ± 0.25	0.21 ± 0.26	6.22 ^***^
Rate of previous no-shows	0.04 ± 0.11	0.05 ± 0.13	0.03 ± 0.1	−14.11 ^***^
Type of patient				443.76 ^***^
New patient	37.25%(28,602)	46.28%(5052)	35.75%(23,550)	
Returned patient	62.75%(48,179)	53.72%(5863)	64.25%(42,316)	
Booking confirmation				1294.47 ^***^
Not confirmed	58.61%(45,005)	74.33%(8113)	56.01%(36,892)	
Confirmed	41.39%(31,776)	25.67%(2802)	43.99%(28,974)	
Type of booking				42.05 ^***^
Contact center	99.8%(76,629)	99.54%(10,865)	99.85%(65,764)	
Web	0.2%(152)	0.46%(50)	0.15%(102)	
*Exogenous factors*				
Day of the week				44.49 ^***^
Monday	15.61%(11,985)	16.76%(1829)	15.42%(10,156)	
Tuesday	16.82%(12,918)	16.56%(1807)	16.87%(11,111)	
Wednesday	16.19%(12,432)	15.72%(1716)	16.27%(10,716)	
Thursday	16.61%(12,751)	16.68%(1821)	16.59%(10,930)	
Friday	16.53%(12,694)	15.62%(1705)	16.68%(10,989)	
Saturday	12.22%(9380)	11.64%(1270)	12.31%(8110)	
Sunday	6.02%(4621)	7.03%(767)	5.85%(3854)	
Month of the year				46.1 ^***^
January	9.72%(7466)	9.73%(1062)	9.72%(6404)	
February	10.34%(7940)	10.93%(1193)	10.24%(6747)	
March	11.34%(8709)	11.74%(1281)	11.28%(7428)	
April	9.69%(7437)	9.69%(1058)	9.68%(6379)	
May	11.23%(8619)	10.43%(1138)	11.36%(7481)	
June	10.15%(7797)	10.46%(1142)	10.1%(6655)	
July	9.74%(7478)	9.69%(1058)	9.75%(6420)	
August	5.58%(4285)	5.34%(583)	5.62%(3702)	
September	9.47%(7270)	8.84%(965)	9.57%(6305)	
October	7.12%(5464)	7.95%(868)	6.98%(4596)	
November	3.55%(2726)	2.99%(326)	3.64%(2400)	
December	2.07%(1590)	2.21%(241)	2.05%(1349)	
Year				83.09 ^***^
2012	28.81%(22,122)	32.24%(3519)	28.24%(18,603)	
2013	34.55%(26,530)	34.08%(3720)	34.63%(22,810)	
2014	36.64%(28,129)	33.68%(3676)	37.13%(24,453)	
Time of the day				186.99 ^***^
6 AM–8 AM	12.02%(9229)	11.66%(1273)	12.08%(7956)	
8 AM-1 PM	35.92%(27,582)	31.54%(3443)	36.65%(24,139)	
1 PM–8 PM	39.46%(30,299)	40.88%(4462)	39.23%(25,837)	
8 PM-2 AM (+1d)	12.6%(9671)	15.91%(1737)	12.05%(7934)	
Long weekend				0.69
No	85.64%(65,753)	85.9%(9376)	85.59%(56,377)	
Yes	14.36%(11,028)	14.1%(1539)	14.41%(9489)	
Weather forecast				0.84
Clear	63.04%(48,406)	62.69%(6843)	63.1%(41,563)	
Rain	24.76%(19,014)	25.1%(2740)	24.71%(16,274)	
Storm	12.19%(9361)	12.2%(1332)	12.19%(8029)	
Text message reminder service				20.54 ^***^
Not yet activated	75.29%(57,806)	76.86%(8389)	75.03%(49,417)	
Activated but not sent	16.04%(12,312)	14.61%(1595)	16.27%(10,717)	
Sent	8.68%(6663)	8.53%(931)	8.7%(5732)	
*Factors associated with the examination*				
No NHS coverage period				0.002
No	90.36%(69,379)	90.34%(9861)	90.36%(59,518)	
Yes	9.64%(7402)	9.66%(1054)	9.64%(6348)	
Contrast agent				31.36 ^***^
No	75%(57,583)	77.15%(8421)	74.64%(49,162)	
Yes	25%(19,198)	22.85%(2494)	25.36%(16,704)	
Price of the examination				6.96 ^***^
Insured Patients	0 ± 0	0 ± 0	0 ± 0	
Other Patients	258.16 ± 117.44	234.99 ± 110.22	261.67 ± 118.11	
Waiting list	7.35 ± 7.72	7.92 ± 9.23	7.25 ± 7.44	−7.13 ^***^
Time allowed	38.43 ± 22.99	38.25 ± 21.29	38.46 ± 23.26	0.35

Descriptive statistics are reported as percentages and number of observations (in parentheses) for categorical variables, as mean ± SD for continuous variables. The last column reports the t-value or the χ^2^-value together with the significance symbols

**Table 3:** Base Levels for the categorical variables included in the regression model

Variable	Base Level
Gender	Male
Age group	46–64
Insurance Status	NHS
Type of patient	New Patient
Booking confirmation	Not confirmed
Type of booking	Contact Center
Day of the week	Wednesday
Month of the year	March
Year	2012
Time of the day	8 AM-1 PM
Long weekend	No
Weather forecast	Clear
Text message reminder service	Not yet activated
No NHS coverage period	No
Contrast agent	No

For the MR ward, descriptive statistics of the variables considered are reported in Table 2 (outliers for variables age and waiting time were removed).

### A statistical model for show/no-show

A logistic regression analysis [19] was used to build a predictive model for a single examination’s no-show *y*_*i*_(*i* = 1, …, *N*) conditional on a vector of explanatory variables *x*_*i*_, with clustered robust standard errors computed to correct for repeated examinations on the same patient:

$$ {\displaystyle \begin{array}{c}{y}_i\mid {x}_i\sim Be\left({\pi}_i\right)\\ {}{\pi}_i=\frac{e^{\eta_i}}{1+{e}^{\eta_i}}\\ {}{\eta}_i={\beta}_0+{\beta}_1{x}_{1,i}+\cdots +{\beta}_k{x}_{k,i}\end{array}} $$

where:

- Be(*π*_*i*_) is a Bernoulli distribution with no-show probability *π*_*i*_;
- *β*_0_, *β*_1_, …, *β*_*k*_ are the regression coefficients;
- *x*_1, *i*_, *x*_2, *i*_, …, *x*_*k*, *i*_ are the explanatory variables (cf. 1).

### An overbooking quasi experiment

To reduce the negative effects caused by non–attendance, starting from January 1, 2015 the healthcare center implemented an overbooking strategy in its MR ward. The reference population included all patients booking an examination from January 1, 2015 to June 31, 2015 for a total of 25,395 patients and 29,320 examinations. During this interval, the average rate of no-show was 14.8%; 68.8% of patients attended all their booked examinations, 12.1% were no-show only once and the remaining 19.1% had more than one no-show. The study included 47.1%, males and 52.9% females with an average age, respectively of 53 and 52.

The correspondent algorithm worked as follows:

1. The lower bound of the 95% Confidence Interval (CI) of the no-show probability was computed (based on the coefficients of the model in section A statistical model for show/no-show) and then multiplied by the theoretical time duration of the booked examination. The results of this product represented the number of expected minutes eligible for overbooking appointments.
2. The expected times computed sub 1. were cumulated across booked appointments and, as soon as the result is equal to or greater than the time needed for that type of examination, an overbooking slot was created and placed next to the existing booking with the highest no-show probability.
3. Such a computational procedure was performed (every evening) 2 days before the day under review and the extra slots were created and then filled (on a first-come first-served basis) with new incoming reservation requests.

A flowchart in Additional file of the paper (Additional file 1: Figure S1) visually represents this procedure.

The opportunity created by its actual implementation in the healthcare center allowed us to verify its effectiveness in a quasi-experiment (i.e. we could not randomize overbooking- and non-overbooking days), comparing the performance relative to days without overbooking.

### An overbooking simulation study

We ran a complementary simulation study in which the overbooking strategy was evaluated under different scenarios to get an evidence less influenced by specific institutional settings and other non-random factors. The simulation code is written in R [20] and is available upon request from the corresponding author.

The study simulated the scheduling procedure of the MR examinations considering the same calendar (January 1 to June 30, 2015) as the overbooking quasi experiment and, for each day, the same number of actual working hours (starting 6 AM). For each booked examination, three random variables were considered:

- the show/no-show behavior (0/1) was simulated through a Bernoulli random variable with probability equal to the no-show probability of the individual evaluated using his/her values of the explanatory variables and the parameters estimated as mentioned in section A statistical model for show/no-show;
- the actual duration of the examination was generated through a gamma random variable whose parameters were estimated from real data (α = 11.73, β = 0.51, E[X] = 22.83; values in minutes);
- the patient early/late arrival (scheduled minus arrival times) was represented by a location-scale Student-T with parameters estimated from real data (μ = − 19.70, σ = 21.43, ν = 4.35; values in minutes).

For sake of simplicity, for each examination we assumed a fixed price of €200 and a fixed theoretical duration of 30′ based on the theoretical duration of the 10 most frequently asked MRs in the healthcare center. Nevertheless, the shorter MR lasts about 15 min, while the longest one takes about 50 min. We also assumed that, for each day, there were enough new requests from patients that could fill the gap generated by no-show. The overbooking algorithm worked exactly as described in section An overbooking quasi experiment.

A first set of simulation experiments considered one MR scanner and different mean levels of no-show (5, 10, 15, 30 and 45%) obtained by adjusting the intercept of our logistic regression model:

$$ {\beta}_0^{\ast }={\beta}_0+\log \left({\overline{p}}_E/\left(1-{\overline{p}}_E\right)\right)-\log \left({\overline{p}}_A/\left(1-{\overline{p}}_A\right)\right) $$

where:

- ${\overline{p}}_E$ is the assumed average probability of no-show (5%, 10%, 15%, 30% or 45%);
- ${\overline{p}}_A$ is the overall probability of no-show in the estimated sample.

A second set of simulations used a fixed 15% mean no-show rate, but a different number of active MR scanners (1 to 5). For each scenario, 100 replications were performed, performing univariate paired t-tests on the equality of the means, with and without overbooking.

## Results

The quasi experiment and the simulation study provided complementary evidences: the former regarded a non-randomized experiment in a specific real setting; the latter concerned a randomized study under different simulated scenarios.

### A statistical model for show/no-show

The main results of the logistic regression analysis are summarized in Table 4. Among the variables included in the category “Patient’s intrinsic factors”, women appeared more likely to no-show than men, as well as patients aged 19–45 and over 80 in comparison to patients in the group 46–64. A higher non–attendance rate was associated with online reservations and with examinations not covered by the NHS, while a lower no-show rate was associated to patients providing a booking confirmation. Previous history was also strongly predictive: patients with higher rates of previous cancellations or no-shows had higher non–attendance rates, while those with previous examinations in the healthcare center had lower propensity to no-show.

**Table 4:** Logistic regression results

Variable		Magnetic Resonance	All wards (average)
Intercept		−1.684 (−42.67)^***^	
*Patient’s intrinsic factors*			
Gender	Female	0.113 (7.62) ^***^	50%
Age group	0–18	−0.003 (− 0.09)	43%
”	19–45	0.105 (6.2) ^***^	71%
”	65–79	−0.118 (−5.39) ^***^	57%
”	80+	0.066 (1.38)	29%
Insurance status	Private	0.371 (4.3) ^***^	100%
Rate of previous cancellations		0.198 (5.82) ^***^	29%
Rate of previous no-shows		1.465 (19.97) ^***^	71%
Type of patient	Returned patient	− 0.45 (−28.27) ^***^	100%
Booking confirmation	Confirmed	−0.98 (−53.38) ^***^	100%
Type of booking	Web	0.933 (9.5) ^***^	29%
*Exogenous factors*			
Day of the week	Monday	0.065 (2.63) ^**^	14%
”	Tuesday	0.04 (1.61)	57%
”	Thursday	0.063 (2.53) ^*^	14%
”	Friday	0.008 (0.32)	0%
”	Saturday	0.085 (3.11) ^**^	43%
”	Sunday	0.337 (9.79) ^***^	50%
Month of the year	January	−0.033 (−0.98)	0%
”	February	0.052 (1.59)	0%
”	April	−0.054 (−1.53)	0%
”	May	−0.087 (−2.63) ^**^	0%
”	June	0.037 (1.1)	14%
”	July	0.001 (0.03)	0%
”	August	−0.029 (−0.74)	0%
”	September	−0.099 (−2.91) ^**^	0%
”	October	0.067 (1.53)	0%
”	November	−0.22 (−3.35) ^***^	14%
”	December	0.046 (0.63)	0%
Year	2013	−0.122 (−6.07) ^***^	17%
”	2014	−0.395 (−12.68) ^***^	29%
Time of the day	6 AM–8 AM	0.165 (6.31) ^***^	100%
”	1 PM–8 PM	0.235 (13.58) ^***^	57%
”	8 PM-2 AM (+1d)	0.507 (20.42) ^***^	100%
Long weekend	Yes	−0.041 (−1.9)	0%
Weather forecast	Rain	0.056 (3.13) ^**^	29%
”	Storm	0.027 (1.23)	0%
Text message reminder service	Activated but not sent	0.063 (1.84)	0%
”	Sent	−0.178 (−4.9) ^***^	67%
*Factors associated with the examination*			
No NHS coverage period	Yes	0.27 (4.6) ^***^	86%
Contrast agent	Yes	0.018 (0.97)	0%
Price of the examination		−0.002 (−7.14) ^***^	50%
Waiting list		0.024 (21.79) ^***^	71%
Time allowed		0.002 (5.7) ^***^	43%
*Goodness of fit measures*		Magnetic Resonance	All wards(average)
R^2^		0.05	0.19
Hosmer-Lemeshow		25.76	15.11
AIC		59,787.9	16,085.55
AUC		0.66	0.77
*Goodness of fit measures – Out of Sample*		Magnetic Resonance	All wards (average)
Hosmer-Lemeshow		38.31	26.52
AUC		0.65	0.75

For the MR ward, estimated parameters, z-value (in parentheses) and significance symbols are reported. For the remaining wards, we reported how many times, in percentage, each variable is significant at α = 0.05. Significance codes: 0’***’ 0.001’**’ 0.01’*’ 0.05

When it came to the “Exogenous factors”, all times of day were positively associated with higher no-show rates when compared to the reference 8 AM-1 PM. During the weekend, there was a higher propensity to non–attendance as well as on days in which the weather forecasts predicted rain. A substantial positive effect came from the introduction of the text message reminder, which generated higher attendance rates in patients receiving it.

Regarding the “Factors associated with the examination”, a higher no-show rate was associated with a longer waiting list (in days) between the booking and the examination day, with longer examinations and with appointments performed when the examination costs were not covered by the NHS. Higher prices of the examination, on the opposite, were related to lower no-show rates: we interpreted this result in the sense that prices are a proxy of the complexity and importance of the service demanded by patients.

As mentioned above, although we detailed results only for the MR ward, we performed analyses for each ward (as reported in the Additional file 1) of the healthcare center. The last column in Table 4 gave the percentage of how many times each variable was a significant predictor of appointment failure (*P* ≤ .05) considering all wards. This allowed us to identify which variables were more relevant for predicting no-shows in a healthcare center with heterogeneous wards, each characterized by its own specificities. The most interesting variables appeared to be age group, insurance status, type of patient (returned or not), booking confirmation and rate of previous no-shows; on the opposite, the rate of previous cancellations was not particularly significant across the wards. About other factors, a significant role was played by time of the day and text message reminder service.

### An overbooking quasi experiment

Considering Table 5, the overbooking strategy (described in section An overbooking quasi experiment) increased the hourly revenues by 15.4%, with a statistically significant difference between the means of the hourly revenue with and without the strategy. The patient’s waiting time increased by 6′12″; staff’s overtime by 10′, while the idle time decreased by 9′06″. Note, however, that the differences between the means of these last three variables in the two subsets were not statistically significant. These results provide evidence that overbooking could conveniently improve the management of the healthcare center, without affecting negatively the quality of the care offered to the patient and the costs of the healthcare center.

**Table 5:** Overbooking quasi experiment. (OB=Overbooking)

Variable	Perc. difference (with - without OB)	Difference (with - without OB)	t-stats
Hourly Revenue (Eur)	15.4	6.9	2.76
Waiting time (Min)	3.66	6.2	0.34
Idle time (Min)	−1.42	−9.1	−1.33
Overtime (Min)	4.05	10	0.3
Number of days with OB		112	
of days without OB		62	

### An overbooking simulation study

Considering the first set of simulations (Table 6), for a mean level of no-show of 15%, the hourly revenue increased by 12.6% with the introduction of the overbooking strategy. Under the same conditions, the patient’s waiting time grew by 3′36″, the overtime increased by 1′30″ (due to longer time needed to complete examinations) and the idle time decreased by 4′06″. The t-tests indicate that the differences between the means of the simulation with and without overbooking were statistically significant for all variables considered.

**Table 6:** Simulation analysis with one scanner and different levels of no-show

	Hourly Revenue (Eur)			Waiting time (Min)			Idle time (Min)			Overtime (Min)		
No-show (%)	Diff. (%)	Average t-stats	Reject (%)	Diff.	Average t-stats	Reject (%)	Diff.	Average t-stats	Reject (%)	Diff.	Average t-stats	Reject (%)
5	2.9	5.76	100	1.2	2.26	56	−0.9	−4.12	100	0.2	0.18	6
10	7.8	11.13	100	2.6	4.85	100	−2.5	−9.19	100	0.2	1.17	7
15	12.6	14.35	100	3.6	6.44	100	−4.1	−12.30	100	0.5	0.44	7
30	26.2	18.87	100	4.9	8.10	100	−9.3	−16.27	100	0.8	0.75	8
45	40.0	20.91	100	4.6	7.33	100	−16.6	−16.97	100	0.9	1.06	18

Reject indicates the percentage of rejections of the t-test at 5%. Difference is computed as with minus without overbooking

By rising the no-show mean level between 5 and 45%, the hourly revenue increased significantly (from 2.9% to 40%), as expected, the waiting time and the overtime remained substantially stable, while the idle time reduced considerably.

Table 7 summarize the second set of simulations (variable number of scanners for a mean no-show probability of 15%); we assumed that all resources were perfectly interchangeable, in the sense that each patient was examined in the first available scanner, not necessarily corresponding to that indicated in the reservation. The hourly revenue gain was stable around 13%, as well as the idle time improvement, approximately constant around 3′36″. The waiting time reduced from 6′42″ to 2′42″, likely as an effect of the interchangeability of the resources. Finally, the effect on the overtime was substantially irrelevant, as indicated by the t-test statistics.

**Table 7:** Simulation analysis with fixed no-show level at 15% and different number of active MR scanners

	Hourly Revenue (Eur)			Waiting time (Min)			Idle time (Min)			Overtime (Min)		
MR scanners	Diff. (%)	Average t-stats	Reject (%)	Diff.	Average t-stats	Reject (%)	Diff.	Average t-stats	Reject (%)	Diff.	Average t-stats	Reject (%)
1	12.4	6.26	100	6.7	7.08	100	−3.3	−12.36	100	0.6	0.79	12
2	13.3	10.32	100	4.4	8.61	100	− 3.5	−18.75	100	0.8	0.47	9
3	13.6	13.30	100	3.5	9.02	100	−3.6	−23.19	100	1.1	0.58	9
4	13.7	15.56	100	3.0	9.66	100	−3.6	−27.34	100	1.3	0.57	12
5	13.8	17.52	100	2.7	9.75	100	−3.6	−30.46	100	2.0	0.78	12

Reject indicates the percentage of rejections of the t-test at 5%. Difference is computed as with minus without overbooking

## Discussion

Patient’s non–attendance to booked examinations has a negative impact on the health system, because individuals who do not show up at booked appointments end up delaying the treatment of other patients, increasing waiting list, wasting resources and reducing the capability of healthcare providers to invest in medical equipment.

The first contribution of this study is in providing additional evidence on the possible predictors of missing medical appointments. Using data coming from a healthcare center located in Southern Italy (with a 14.6% average level of no-show, consistent with [2, 3]) we built a statistical model to predict non–attendance. To this aim, we classified each explanatory variable as a “Patient’s intrinsic factor”, an “Exogenous factor” or a “Factor associated with the examination”. This classification represented a first useful contribution of this study because, for example, the healthcare center may reduce no-shows working on the factors it can manage (for example, reminder text messages), since it has little control on patient’s intrinsic characteristics, factors associated with the examination or on the weather.

Regarding the structural characteristics of the patients, young adults represented the category more likely to miss scheduled appointments [6, 7, 10]. About the exogenous factors, no-show was related to the time of the day, day of the week and weather conditions [16]. The patient’s previous history was strongly predictive: individuals with previous cancellations or no-shows had higher non–attendance rates [2, 7, 21], while those with one or more previous examinations in the healthcare center had lower propensity to no-show [9]. As in previous studies, we found that non–attendance was associated with the waiting list for an appointment (days between booking and the examination) [6, 7]. Examinations booked many days in advance were more likely to be forgotten or to be not useful anymore (maybe because, in the meantime, the health issue had disappeared or, rather, patients required hospitalization). We also found a higher no-show rate associated to online reservations and to examinations not covered by the NHS (or performed during a period not covered by the NHS). This finding relates to the specific regional healthcare system, but it suggests the more general idea that, when patients had to pay for their examinations, they tended to miss the appointments more often than when the NHS paid for the service. We do not have an explanation as of why appointments booked online were more likely to be missed. We could speculate that patients booking online figure that, if they did not show up for their examination, they could easily book a new appointment without having to talk to a real person, who might have known that the patient had not shown up to a previous appointment. Moreover, the online system used by the healthcare center shows the patient all the available slots, while the call center’s staff proposes only a few slots to the patient, offering more only upon a patient’s request. Of course, the significant patient’s intrinsic factors are only under limited control by healthcare providers. Big healthcare providers or payers, such as integrated managed care organizations or the NHS, may try to implement educational campaigns to raise the patients’ awareness about the negative impact of missed appointments.

Among exogenous factors, consistently with [4], our results suggest that no-show rates can vary according to time-related factors: we could speculate that, in some hours of the day (i.e. before 6 AM and after 8 PM), public transportation may be less frequent or even not available at night or in the weekends.

A positive effect came from some expedients used to reduce missed appointments: a lower propensity to no-show was found for patients answering to a phone call or receiving a text message reminder [5, 8–11]. This finding strengthens the existing evidence that “text messaging reminders increased attendance at healthcare appointments compared to no reminders, or postal reminders” [22]. This is an easy strategy to reduce the number of no-shows due to patients’ forgetfulness and it should be implemented in most outpatient centers. Web, mobile and, in general, self-service appointment possibilities, in fact, are important technological opportunities, which may be labor-efficient for the provider organization as well as convenient for the patient. The no-show rate in the period without text message reminder is 14.85% while the non attendance rate in the period with it is 13.80%. As a consequence, if text message reminder was not activated, the overbooking strategy would give an even greater contribution in the management of no-show (according to the results of the simulation experiment in section An overbooking simulation study) because there will be an higher non attendance rate.

Among factors associated with the examination, both the price and the time allowed by the healthcare center had a small but significant impact on patient’s no-show. The tendency to show less often for more expensive examinations may be due to the fact that more expensive procedures may be more important for the patients. For instance, price may be a proxy for the importance of the examination because doctors may tend to prescribe MRs only if in the lack of alternative cheaper diagnostics. As of the positive impact of longer MR procedures, it may be related to the higher anticipated stress, the longer the time spent in a MR scanner.

Our predictive model (independent variables, magnitude and significance of the corresponding coefficients) cannot be piece-by-piece transposed to different socio-economical contexts and health systems. However, we show that is possible to reliably predict no-shows using context-specific patient’s intrinsic factors, factors associated with the examination and exogenous factors. Prediction models built with local data can then be used to develop context-specific overbooking algorithms.

Another contribution of this study is the evaluation of the overbooking strategy built on our no-shows predictors. When not able to intervene on non-attendance causes, the center may resort to overbooking to manage its effects. It represents a trade-off between the provider’s productivity and the waiting list for a new appointment on the one hand, and overcrowding and delays due to patients showing up in larger numbers than expected at a given time of day [4], on the other. We contribute to the previous literature on overbooking in healthcare [4, 14–16] by testing in a real setting a strategy, which used a fairly complex predictive model. We showed a significant productivity gain (+ 15.4%) in terms of hourly revenue, increasing delays suffered by patients only by non-significant amounts. This suggests that overbooking could improve healthcare centers and, indirectly, the quality of service.

We speculate that the negligible effects of overbooking on patients were due to:

- the quality of the underlying predictive model, which took into account patient’s intrinsic factors, exogenous factors and other characteristics associated with the examination and was based on a considerable amount of observations;
- the fact that the predictive model was built on context-specific data;
- the size of this healthcare center operations, with five MR scanners working alongside one another.

In order to complement these findings with a more general evidence, we also proposed a simulation study considering various scenarios across several numbers of active MR scanners and rates of no-shows. The results of the simulations are in line with [4], demonstrating that overbooking strategies can increase clinic productivity, reduce resource idle time, although they may increase patient’s waiting time and staff’s overtime. An important policy implication is that the increase of the number of active MR scanners, for the same average level of no-shows, may improve the efficiency of the healthcare center.

Regarding the limitations, our analysis was performed on just one healthcare center and, accordingly, it is not necessarily portable to others. Moreover, such a healthcare center operates within the Italian NHS, whose budget covers most medical examinations. This underlines the importance of estimating model parameters on variables and data reflecting the specific organizational options in each healthcare center. A second limitation when considering several active MR scanners: we assumed that the patient was diagnosed with the first resource available, while, in other settings, one encounters technical limits to substitutability across scanners (and hence a need to stick to the one listed in the reservation). This case was not explored in our simulation study.

The overbooking procedure is dictated by an organizational choice by the center: more frequent updates may strain the management of the high incoming phone call flow to book an examination. Further refinements could come from the availability of patients’ socioeconomic conditions, levels of education, distance to reach the healthcare center, which could be significant for the prediction of the no-show [2, 5–7]. Moreover, clinical information about the patients could signal the urgency of the examination and/or the possibility of selecting homogeneous patient cohorts.

## Conclusions

This study contributed to the literature on appointment attendance and overbooking in healthcare by showing, in a setting with real data, that overbooking may imply benefits with limited side effects.

Moreover, we strengthened the evidence of a predictive model testing most of the variables considered by previous studies in a single model, using a large dataset in a specific socio-economic context. This predictive model also introduced two new variables influencing no-show: online booking and insurance/NHS coverage. This study provided an encouraging evidence that overbooking procedures could improve the management of healthcare centers. The current upward trend regarding the availability of data and the access to complex data analysis tools, allows an increasing number of healthcare organizations to adopt overbooking practices based on well performing predictive models, such as that derived in this paper. The quasi experiment gave real setting-evidence that overbooking could improve efficiency - and, indirectly, the quality of services - while increasing waiting time suffered by patients only by small and statistically non-significant amounts. This evidence encourages further studies to strengthen the evidence about an extended adoption of overbooking strategies in healthcare centers.

## Additional file

Additional file 1: Web appendix with the logistic regression results for the other wards (Echography, Echo Doppler, Mammography, Orthopantomography, Radiography, Computer-Assisted Tomography) and the description and the representation of the flow chart. (PDF 668 kb)

## Abbreviations

MR: Magnetic resonance
NHS: National health service
OB: Overbooking
