# Supplementary material for: Using overbooking to manage no-shows in an Italian healthcare center
Source: BMC Health Serv Res. 2018 Mar 15;18:185. doi: 10.1186/s12913-018-2979-z (PMC5856203; doi:10.1186/s12913-018-2979-z)
Supplement: Supplementary file 1 — Web appendix with the logistic regression results for the other wards (Echography, Echo Doppler, Mammography, Orthopantomography, Radiography, Computer-Assisted Tomography) and the description and the representation of the flow chart. (PDF 668 kb) [file 12913_2018_2979_MOESM1_ESM.pdf]

Additional file 1: Web appendix

Table 1: Logistic regression results for the Echography ward (2 available scanners).

Variable		Echography
Intercept		0.563 (4.94) ***
Patient's intrinsic factors		
Gender	Female	0.076 (1.93)
Age group	0-18	0.349 (4.69) ***
"	19-45	0.191 (4.34) ***
"	65-79	0.031 (0.46)
"	80+	0.116 (0.94)
Insurance status	Private	-1.726 (-21.81) ***
Rate of previous cancellations		0.179 (1.95)
Rate of previous no-shows		1.796 (10.32) ***
Type of patient	Returned patient	-0.781 (-18.36) ***
Booking confirmation	Confirmed	-1.578 (-19.19) ***
Type of booking	Web	-0.015 (-0.06)
Exogenous factors		
Day of the week	Monday	-0.118 (-1.86)
"	Tuesday	-0.164 (-2.74) **
"	Thursday	-0.228 (-3.59) ***
"	Friday	-0.038 (-0.7)
"	Saturday	-0.405 (-4.89) ***
Month of the year	January	-0.095 (-1.01)
"	February	-0.029 (-0.24)
"	April	-0.046 (-0.47)
"	May	0.179 (1.85)
"	June	0.197 (1.97) *
"	July	-0.059 (-0.58)
"	August	0.258 (2.28) *
"	September	-0.168 (-1.72)
"	October	0.157 (1.33)
"	November	-0.029 (-0.2)
"	December	0.062 (0.41)
Year	2013	-0.006 (-0.11)
"	2014	-0.127 (-1.5)
Time of the day	1PM-8PM	-0.094 (-2.31) *
Long weekend	Yes	0.082 (1.46)
Weather forecast	Rain	0.148 (3.27) **
"	Storm	0.056 (0.93)
Text Message Reminder Service	Activated but not sent	-0.08 (-0.86)
"	Sent	-0.401 (-4.06) ***
Factors associated with the examination		
No NHS coverage period	Yes	0.296 (2.79) **
Price of the examination		0.001 (3.08) **
Waiting list		0.007 (3.95) ***
Time allowed		-0.002 (-1.96)
Goodness of fit measures		Echography
	R^2	0.1
	Hosmer-Lemeshow	16.3
	AIC	10169.02
	AUC	0.72
Goodness of fit measures - Out of Sample		Echography
	Hosmer-Lemeshow	57.13
	AUC	0.68

Estimated parameters, z-value (in parentheses) and significance symbols are reported.
Significance codes: 0 '***' 0.001 '**' 0.01 '*' 0.05

Table 2: Logistic regression results for the Echo Doppler ward (2 available scanners).

Variable		Echo Doppler
Intercept		1.403 (106)
Patient's intrinsic factors		
Gender	Female	-0.28 (-1.75)
Age group	0-18	-0.345 (-0.86)
"	19-45	0.062 (0.36)
"	65-79	0.018 (0.09)
"	80+	-0.312 (-0.83)
Insurance status	Private	-1.766 (-3.13) **
Rate of previous cancellations		0.295 (0.8)
Rate of previous no-shows		1.416 (2.27) *
Type of patient	Returned patient	-1.141 (-6.99) ***
Booking confirmation	Confirmed	-1.369 (-8.03) ***
Type of booking	Web	1.372 (1.13)
Exogenous factors		
Day of the week	Monday	-0.093 (-0.31)
"	Tuesday	-0.687 (-1.3)
"	Thursday	-1.269 (-2.44) *
"	Friday	-0.92 (-3.49) ***
"	Saturday	0.342 (0.42)
Month of the year	January	0.035 (0.08)
"	February	0.448 (1.18)
"	April	0.464 (0.84)
"	May	0.759 (1.45)
"	June	0.473 (0.89)
"	July	0.226 (0.41)
"	August	0.374 (0.53)
"	September	0.436 (0.78)
"	October	0.056 (0.1)
"	November	-0.124 (-0.18)
"	December	0.575 (0.86)
Year	2013	0.5 (1.29)
"	2014	0.876 (1.54)
Time of the day	1PM-8PM	-0.116 (-0.47)
Long weekend	Yes	0.289 (1.26)
Weather forecast	Rain	-0.41 (-2.12) *
"	Storm	-0.014 (-0.06)
Text Message Reminder Service	Activated but not sent	-0.691 (-1.41)
"	Sent	-0.862 (-1.7)
Factors associated with the examination		
No NHS coverage period	Yes	0.326 (0.85)
Price of the examination		-0.001 (-0.69)
Waiting list		0.013 (1.5)
Time allowed		-0.013 (-1.46)

Goodness of fit measures		Echo Doppler
	R^2	0.13
	Hosmer-Lemeshow	7.58
	AIC	779.09
	AUC	0.74

Goodness of fit measures - Out of Sample		Echo Doppler
	Hosmer-Lemeshow	19.22
	AUC	0.62

Estimated parameters, z-value (in parentheses) and significance symbols are reported.
Significance codes: 0 '***' 0.001 '**' 0.01 '*' 0.05

Table 3: Logistic regression results for the Mammography ward (1 available scanner).

Variable		Mammography
Intercept		0.494 (1.11)
Patient's intrinsic factors		
Age group	19-45	0.021 (0.15)
"	65-79	-0.879 (-5.49) ***
"	80+	-1.382 (-2.33) *
Insurance status	Private	-4.885 (-6.15) ***
Rate of previous cancellations		0.307 (1.18)
Rate of previous no-shows		1.72 (2.41) *
Type of patient	Returned patient	-1.433 (-9.35) ***
Booking confirmation	Confirmed	-1.645 (-11.62) ***
Type of booking	Web	1.065 (1.98) *
Exogenous factors		
Day of the week	Monday	0.257 (1.36)
"	Tuesday	0.615 (3.28) **
"	Thursday	0.237 (1.27)
"	Friday	0.244 (1.32)
"	Saturday	-0.126 (-0.43)
Month of the year	January	-0.3 (-1.09)
"	February	0.304 (1.14)
"	April	-0.1 (-0.4)
"	May	0.029 (0.1)
"	June	0.664 (2.5) *
"	July	0.18 (0.69)
"	August	0.34 (1.05)
"	September	0.169 (0.52)
"	October	0.041 (0.11)
"	November	-0.275 (-0.63)
"	December	-0.059 (-0.11)
Year	2013	0.19 (0.88)
"	2014	0.209 (0.6)
Time of the day	1PM-8PM	0.244 (1.33)
Long weekend	Yes	0.012 (0.07)
Weather forecast	Rain	-0.032 (-0.21)
"	Storm	-0.166 (-0.97)
Text Message Reminder Service	Activated but not sent	-0.687 (-2.27) *
"	Sent	-0.558 (-1.69)
Factors associated with the examination		
No NHS coverage period	Yes	4.185 (5.73) ***
Price of the examination		-0.015 (-1.72)
Waiting list		0.017 (4.2) ***
Time allowed		0.004 (0.38)
Goodness of fit measures		Mammography
	R^2	0.39
	Hosmer-Lemeshow	10.54
	AIC	1480.78
	AUC	0.9
Goodness of fit measures - Out of Sample		Mammography
	Hosmer-Lemeshow	13.84
	AUC	0.88

Estimated parameters, z-value (in parentheses) and significance symbols are reported.
Significance codes: 0 '***' 0.001 '**' 0.01 '*' 0.05

Table 4: Logistic regression results for the Orthopantomography ward (1 available scanner).

Variable		Orthopantomography
Intercept		-0.103 (-0.21)
Patient's intrinsic factors		
Gender	Female	-0.018 (-0.17)
Age group	0-18	0.291 (1.68)
"	19-45	0.46 (3.79) ***
"	65-79	-0.79 (-4.86) ***
"	80+	-1.017 (-3.02) **
Insurance status	Private	-3.945 (-22.77) ***
Rate of previous cancellations		0.402 (1.68)
Rate of previous no-shows		0.314 (0.81)
Type of patient	Returned patient	-0.774 (-6.44) ***
Booking confirmation	Confirmed	-2.162 (-8.07) ***
Type of booking	Web	-0.484 (-1.25)
Exogenous factors		
Day of the week	Monday	-0.18 (-1.05)
"	Tuesday	-0.477 (-2.75) **
"	Thursday	-0.29 (-1.65)
"	Friday	-0.307 (-1.85)
"	Saturday	0.053 (0.29)
Month of the year	January	0.148 (0.4)
"	February	0.286 (0.79)
"	April	-0.201 (-0.5)
"	May	-0.155 (-0.4)
"	June	-0.074 (-0.19)
"	July	-0.179 (-0.46)
"	August	-0.225 (-0.52)
"	September	-0.092 (-0.23)
"	October	0.408 (0.94)
"	November	0.443 (0.96)
"	December	0.489 (1.03)
Year	2014	-0.535 (-1.36)
Time of the day	1PM-8PM	0.428 (3.7) ***
Long weekend	Yes	0.044 (0.28)
Weather forecast	Rain	-0.023 (-0.17)
"	Storm	0.065 (0.46)
Text Message Reminder Service	Activated but not sent	0.555 (1.36)
Factors associated with the examination		
No NHS coverage period	Yes	1.844 (6.04) ***
Waiting list		0.046 (4.63) ***
Time allowed		0.05 (2.01) *

Goodness of fit measures		Orthopantomography
	R^2	0.31
	Hosmer-Lemeshow	9.98
	AIC	1589.04
	AUC	0.86

Goodness of fit measures - Out of Sample		Orthopantomography
	Hosmer-Lemeshow	10.22
	AUC	0.9

Estimated parameters, z-value (in parentheses) and significance symbols are reported.
Significance codes: 0 '***' 0.001 '**' 0.01 '*' 0.05

Table 5: Logistic regression results for the Radiography ward (1 available scanner).

Variable		Radiography
Intercept		-0.063 (-0.65)
Patient's intrinsic factors		
Gender	Female	0.125 (3.42) ***
Age group	0-18	0.227 (3.61) ***
"	19-45	0.368 (7.67) ***
"	65-79	-0.596 (-11.86) ***
"	80+	-0.51 (-6.03) ***
Insurance status	Private	-3.772 (-43.26) ***
Rate of previous cancellations		0.132 (1.62)
Rate of previous no-shows		1.806 (10.86) ***
Type of patient	Returned patient	-0.808 (-19.24) ***
Booking confirmation	Confirmed	-1.48 (-30.76) ***
Type of booking	Web	0.536 (1.52)
Exogenous factors		
Day of the week	Monday	0.033 (0.55)
"	Tuesday	0.046 (0.78)
"	Thursday	0.03 (0.51)
"	Friday	0.104 (1.72)
"	Saturday	0.288 (4.45) ***
Month of the year	January	-0.064 (-0.75)
"	February	0.063 (0.77)
"	April	0.139 (1.55)
"	May	0.014 (0.16)
"	June	-0.026 (-0.29)
"	July	-0.175 (-1.92)
"	August	-0.062 (-0.63)
"	September	0.088 (0.82)
"	October	0.127 (1.12)
"	November	-0.192 (-1.36)
"	December	-0.142 (-0.99)
Year	2013	0.016 (0.32)
"	2014	-0.075 (-0.98)
Time of the day	1PM-8PM	0.186 (4.58) ***
Long weekend	Yes	-0.046 (-0.91)
Weather forecast	Rain	-0.016 (-0.37)
"	Storm	0.05 (0.89)
Text Message Reminder Service	Activated but not sent	0.023 (0.25)
"	Sent	-0.573 (-5.73) ***
Factors associated with the examination		
No NHS coverage period	Yes	2.651 (20.94) ***
Price of the examination		0 (0.97)
Waiting list		0.031 (10.38) ***
Time allowed		-0.004 (-2.42) *
Goodness of fit measures		Radiography
	R^2	0.25
	Hosmer-Lemeshow	20.18
	AIC	12764.35
	AUC	0.84
Goodness of fit measures - Out of Sample		Radiography
	Hosmer-Lemeshow	31.13
	AUC	0.85

Estimated parameters, z-value (in parentheses) and significance symbols are reported.
Significance codes: 0 '***' 0.001 '**' 0.01 '*' 0.05

Table 6: Logistic regression results for the Computer-Assisted Tomography ward (2 available scanners).

Variable		Computer-Assisted Tomography
Intercept		-1.639 (-26.35) ***
Patient's intrinsic factors		
Gender	Female	0.15 (6.38) ***
Age group	0-18	0.244 (3.8) *
"	19-45	0.155 (5.01) ***
"	65-79	-0.043 (-1.46)
"	80+	0.137 (2.71) **
Insurance status	Private	-0.319 (-3.73) **
Rate of previous cancellations		0.259 (4.65) ***
Rate of previous no-shows		1.677 (15.37) ***
Type of patient	Returned patient	-0.748 (-29.22) ***
Booking confirmation	Confirmed	-1.056 (-36) ***
Type of booking	Web	1.049 (4.93) ***
Exogenous factors		
Day of the week	Monday	0.113 (2.92) **
"	Tuesday	0.114 (3.01) **
"	Thursday	0.039 (0.98)
"	Friday	-0.022 (-0.54)
"	Saturday	0.018 (0.39)
"	Sunday	0.135 (1.02)
Month of the year	January	0.136 (2.68) **
"	February	0.054 (1)
"	April	-0.033 (-0.59)
"	May	0.007 (0.13)
"	June	-0.134 (-2.43)
"	July	-0.063 (-1.16)
"	August	0.111 (1.73)
"	September	0.02 (0.37)
"	October	0.104 (1.49)
"	November	-0.088 (-0.78)
"	December	0.014 (0.12)
Year	2013	-0.026 (-0.83)
"	2014	-0.36 (-7.57) ***
Time of the day	6AM-8AM	0.192 (3.78) ***
"	1PM-8PM	0.111 (4.01) ***
"	8PM-2AM (+1d)	0.524 (8.63) ***
Long weekend	Yes	0.095 (2.79) **
Weather forecast	Rain	0.019 (0.65)
"	Storm	-0.037 (-1.02)
Text Message Reminder Service	Activated but not sent	-0.088 (-1.73)
"	Sent	-0.276 (-4.99) ***
Factors associated with the examination		
No NHS coverage period	Yes	0.855 (8.18) ***
Contrast agent	Yes	0.014 (0.51)
Price of the examination		-0.001 (-5.91) ***
Waiting list		0.004 (2.42) *
Time allowed		-0.001 (-1.43)

Goodness of fit measures		Computer-Assisted Tomography
	R^2	0.07
	Hosmer-Lemeshow	15.42
	AIC	26028.66
	AUC	0.7

Goodness of fit measures - Out of Sample		Computer-Assisted Tomography
	Hosmer-Lemeshow	15.79
	AUC	0.67

Estimated parameters, z-value (in parentheses) and significance symbols are reported.
Significance codes: 0 '***' 0.001 '**' 0.01 '*' 0.05

Description of the flow chart

Main procedure:

- The starting block (A) declares/reads the needed variables.
- Blocks from (B) to (G) are cycled a number of times equal to the number of days in `DaysList`. The generic day is denoted with `d`.
- Block (B) :
  - draws randomly the set of patients of the day (`DayPatients`)
  - builds the set of independent variables (`IndepVars`) of the patients selected by appending to their characteristics the variables related to the calendar of the examinations
  - computes the corresponding vector of no-show probabilities using `Coef` and `IndepVars`
- In case overbooking is active, the overbooking procedure is executed (C) and the corresponding overbooked patients (in terms of their no-show probability) are appended to the set of patients of the day (D).
- Block (E) computes the theoretical starting time for each examination (`ExaminationsBegin`) of day `d` to be passed to the next block.
- Block (F) calls the procedure that simulates the activity of the center in day `d`, delivering the information needed to compute the daily statistics (G).

Overbooking procedure:

- The starting block (A) reads the input variables from the main procedure.
- Block (B) computes the theoretical minutes eligible for overbooking (`OEM`) for each patient of the day, computes the number of patients (`NPatients`), and initializes to zero the minutes eligible for overbooking (`Sum`) and the number of extra-slots (`ExtraSlots`).
- Blocks (C) and (D) are cycled a number of times equal to `NPatients`.
- At the end, the number of `ExtraSlots` of the day is returned to the main procedure.

SimVar procedure:

- The starting block (A) reads the input variables from the main procedure.
- Block (B) computes the number of patients and initializes to zero some variables incremented in the cycle.
- Blocks (C) and (D) are cycled a number of times equal to the number of patients in the day to get the simulated number of show patients (`NShowPatients`), the finish time of the last patient (`FinishTime`) and the total waiting and idle times (`WaitingTime` and `IdleTime`, respectively).
- At the end (E), the following output is returned to the main procedure: the number of show patients (`NShowPatients)`, the finish time of the last show patient (`FinishTime`), and the average idle and waiting time (`IdleTime` and `WaitingTime).`

Figure 1: Flowchart of the overbooking algorithm – Main Procedure.

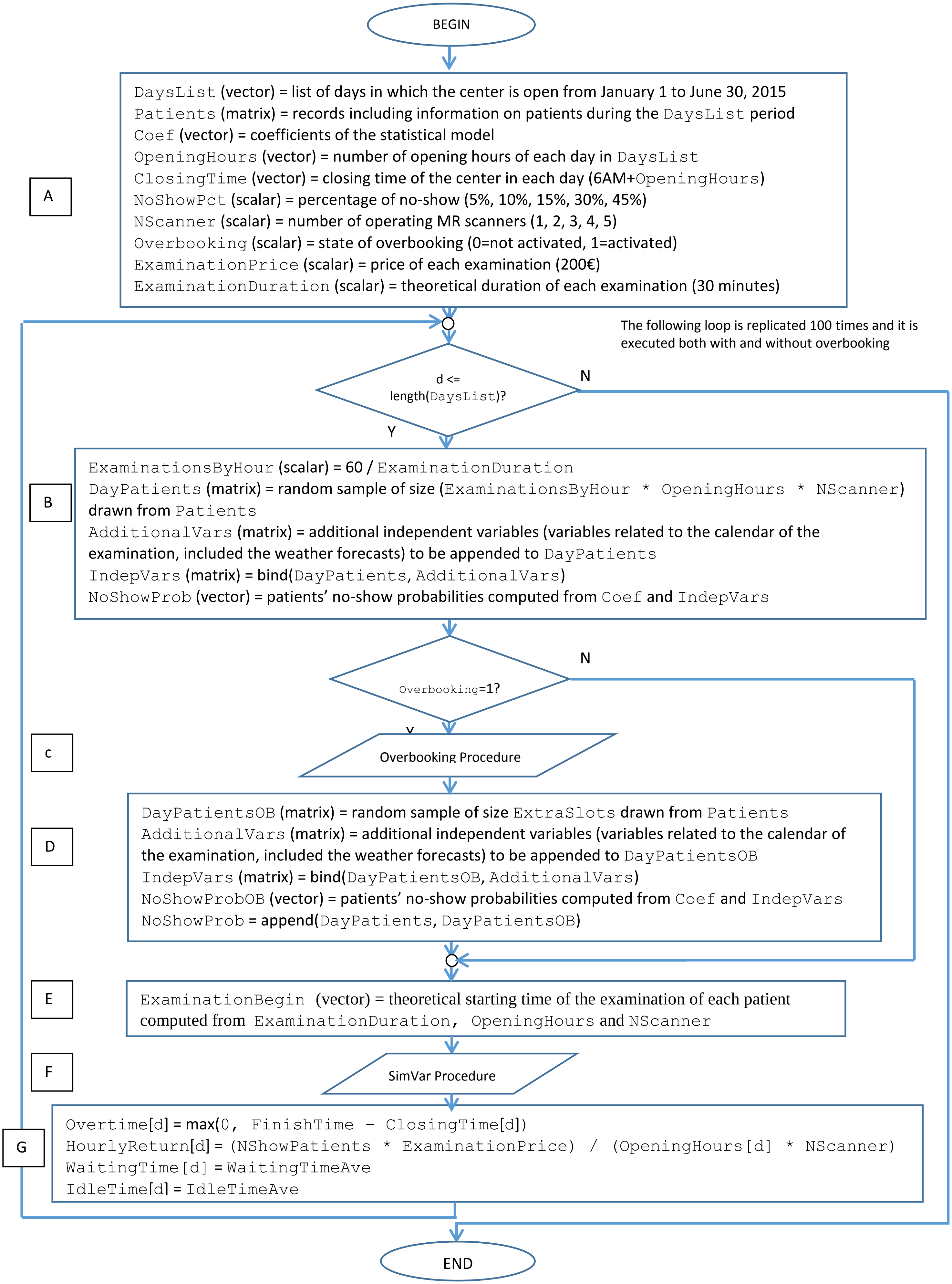

Figure 2: Flowchart of the overbooking algorithm – Overbooking Procedure.

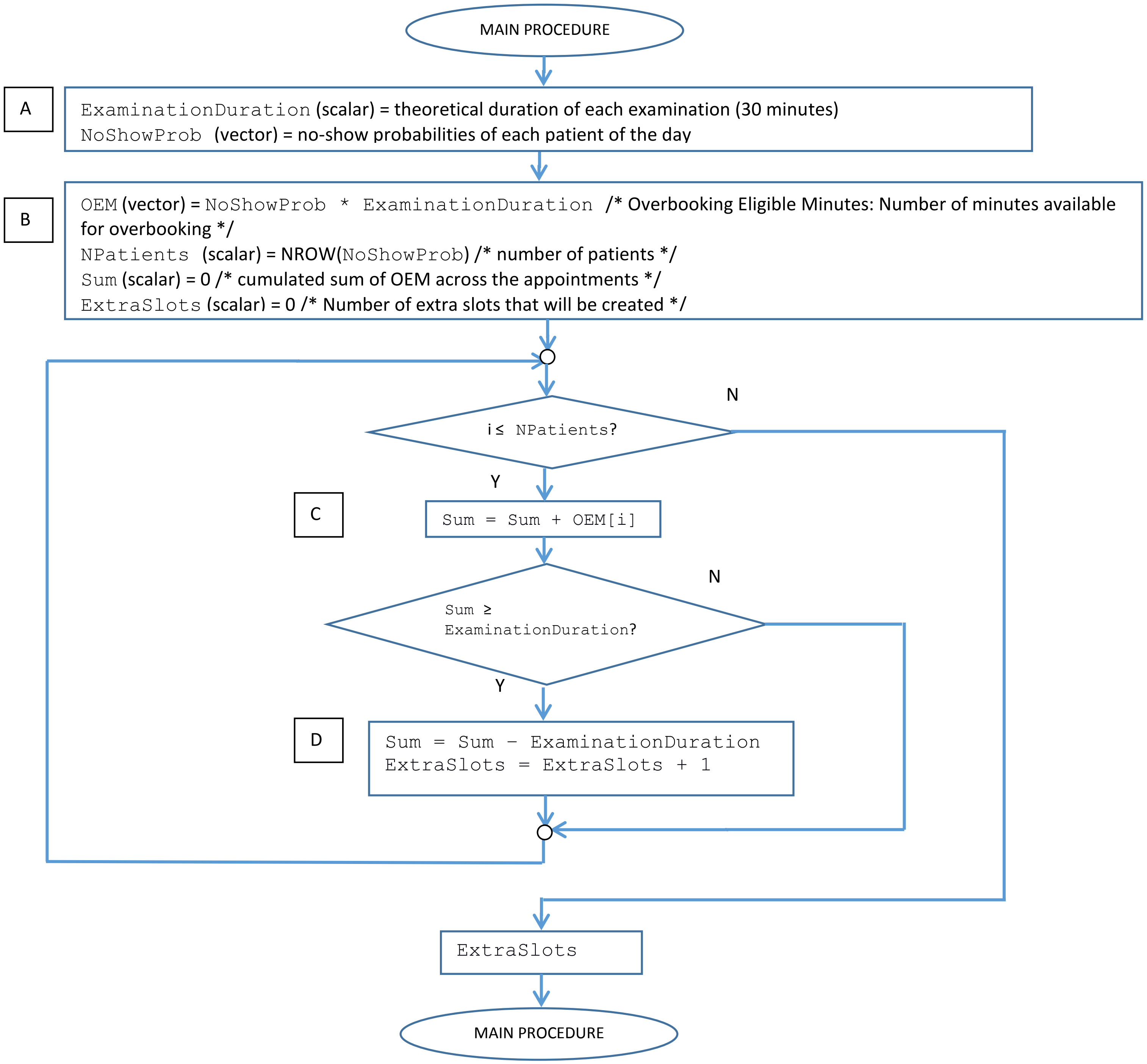

Figure 3: Flowchart of the overbooking algorithm – SimVar Procedure.

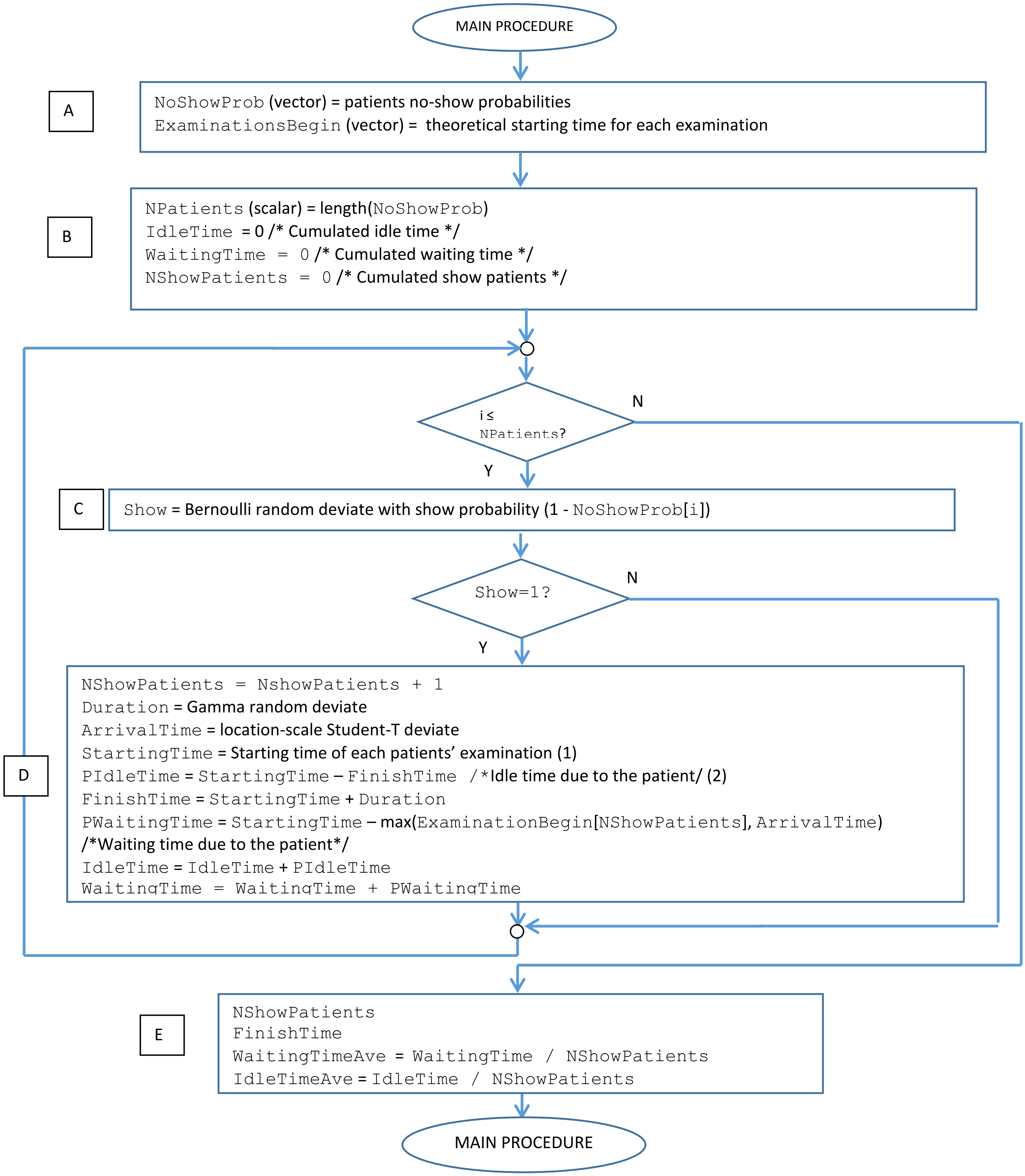

(1) It is computed with a rule that takes into account the arrival time of the current patient as well as the advance/late arrival and the duration of the examination of the previous patients

(2) It depends on the starting time of its examination as well as the finish time of the previous patient. For the first patient, this variable is 0, otherwise its value depends on the patient's delay
